# Nutritional Evaluation of Beetroots (*Beta vulgaris* L.) and Its Potential Application in a Functional Beverage

**DOI:** 10.3390/plants9121752

**Published:** 2020-12-10

**Authors:** Eman Abdo, Sobhy El-Sohaimy, Omayma Shaltout, Ahmed Abdalla, Ahmed Zeitoun

**Affiliations:** 1 Faculty of Agriculture (Saba Basha), Alexandria University, Alexandria 21531, Egypt; eman-abdo@alexu.edu.eg (E.A.); prof.dr.Omimaelsaidshaltout@alexu.edu.eg (O.S.); prof.dr.Ahmedelsaidmohmedabdallah@alexu.edu.eg (A.A.); zaytoun19@alexu.edu.eg (A.Z.); 2 Department of Technology and Organization of Public Catering, Institute of Sport Tourism and Service, South Ural State University, 454080 Chelyabinsk, Russia; 3 Department of Food Technology, Arid Lands Cultivation Research Institute, City of Scientific Research and Technological Applications, New Borg El Arab 21934, Alexandria, Egypt

**Keywords:** Beetroot (*Beta vulgaris* L.), juice, bioactive components, fermented beverage, probiotics

## Abstract

Beetroot is a good source of minerals, fibers, and bioactive components. The present research work was conducted to evaluate the nutritional quality of beetroots (juice, peels, leaves and pomace) enhancing the extracted bioactive components, and developing a functional probiotic beverage. Chemical composition and minerals content of beetroot parts were estimated. The bioactive components were extracted by instant extraction method (IEM) and overnight extraction method (at −20 °C) (OEM) to determine total phenolics, flavonoids, and DPPH inhibition ratio. The extracted beetroot juice was mixed with milk for valorization of the beverage nutritional value and fermented with LA-5 and ABT-5 cultures to create a novel functional beverage. Chemical composition, minerals content, and bioactive components of beverages were estimated. The leaves exhibited the highest calcium content (1200 mg/100 g). Juice showed the highest amount of all minerals except for calcium and magnesium. Overnight extraction method (OEM) increased the antioxidant activity in peels and stems. Natural juice exhibited the highest activity compared to extracts. Fermentation of beet-milk beverage with LA-5 and ABT-5 cultures enhanced the beverage taste, flavor, and antioxidant capacity. Beetroot wastes and juice comprise a valuable nutritional source. Fermentation improved the nutritional value of beetroot and the acceptability of the product.

## 1. Introduction

During the latest decades, the humans’ awareness regarding the importance of vegetable consumptions elevated with believe that vegetables and fruits are a rich source of bioactive components which confirmed their participation in health improvement rather than the use of supplements [1,2]. Accordingly, the production of vegetables increased worldwide significantly from 682.43 million tons in 2000 to 1088.9 million tons in 2018 [1,2,3]. Consequently, root and tuber vegetable production raised from 8.99 million tons in 2008 to 10.53 million tons in 2018 worldwide, where Egypt ranked as the first producer country in North Africa by nearly 5221 tons [4]. Beetroot (*Beta vulgaris* L.) is an herbaceous biennial plant classified as one of the *Chenopodiaceae* family. The taproot found either in yellow pulp color or red [5,6,7] where the red root utilized in salad, juice, food coloring, and as a medicine [6,8] that emerged along the Mediterranean coast. Beets are considered as one of the most effective vegetables, they are a source of betalain pigment in addition to phenolic acids such as gallic, syringic, and caffeic acids and flavonoids. It has anti-inflammatory, and antioxidant effects, which scavenge free radical from the cells promoting cancer prevention by inhibiting the tumor cells proliferation, reducing the risk of cardiovascular diseases, and expelling kidney stones [5,6]. Studies also revealed that it reduced the low-density lipoprotein (LDL) oxidation by 50% [9], decline the blood glucose after beetroot consumption by 40% [10]. Beetroot also considered as a good source of minerals such as iron, calcium, phosphorus, potassium, sodium, and zinc, in addition to vitamins like biotin, niacin, folate [11]. Exposure of beetroot to thermal processing increases the loss and the degradation of vitamins, minerals, and bioactive components, resulting in a significant reduction in the concerning health benefits. Fermentation of beetroot by lactic acid bacteria aids in the prevention of bioactive components degradation, where fermented beetroot juice retained its antimutagenic effect for 30 days under refrigerating [12]. Juice is a good source of carbohydrates, which is a suitable medium for homo-fermentation by probiotic strains [13] such as *Lactobacillus acidophilus*, *Lactobacillus plantarum*, *Lactobacillus casei*, and *Bifidobacterium bifidum* [14]. During fermentation, lactic acid bacteria produce vitamins that have the ability to enhance the nutritional value of the products [13], and lactic acid that is responsible for lowering the pH of the juice thus reducing the growth of the spoilage microorganisms [15], thus prolonging the shelf life [13]. The globally widespread of fermented beverages could be due to its health properties; as lactic acid bacteria decrease the B-glucuronidase activity which resulted in the prevention of cancer, particularly colon cancer besides, the reduction of the pathogens, and it is also proper for the lactose-intolerance people [14]. Additionally, studies revealed that probiotics could enhance immunity and participate in memory impairment [16]. Incorporating beetroot juice in the production of fermented product could affect the human’ health positively, as the phytochemicals can bind the carcinogens reducing their transferability into the cell, thus preventing the cellular DNA mutation [12]. Extraction of the juice from the beetroot plant resulted in extra peels and pomace, in addition to the removed stems and leaves wastes. The residues from processing of the beetroots like peels, seeds, stems, and pomaces reached up to 1.3 billion tons yearly as estimated by FAO which represented one-third of food industry production [17]. Those wastes were discarded for a long time by manufacturers or used as animal feed, or fertilizers until recent studies showed that they are a valuable source of bioactive components which could be used as food additives or formulating novel functional foods [17,18]. Furthermore, those by-products exhibited a significant anti-microbial effect compared to the influence of synthetic antibiotics [19,20]. Beet peels showed a high antioxidant activity because it contains the highest betalain content compared to the other parts [9,19,20]. Like peels, the beet pulp revealed an extraordinary antioxidant effect due to its significant amount of betalain, as well as the presence of other phenolic compounds like “ferulic, vanillic, p-hydroxybenzoic, caffeic, and catechuic acid [19,20]. Leaves are an impressive nutritive source as they contain a tremendous concentration of polyunsaturated fatty acids particularly, alpha-linolenic acid. However, it is cut off from pulps and discarded, mostly because of the dietary habits and the low information about its health benefits [8]. In Egypt, limited studies were carried out to determine the nutritional quality of beetroot peels and pomaces resulting from the juice extraction, stems, and leaves. Therefore, the current study was conducted to evaluate the nutritional quality of the beetroots to ascertain its potential use in food technology. Furthermore, explore the effect of using the probiotic strains [LA5 strain (*Lactobacillus acidophilus*), and ABT5 strain which consist of (*Lactobacillus acidophilus*, *Bifidobacterium bifidum*, and *Streptococcus thermophilus*)] on the nutritional quality and sensory attributes of beetroot juice mixed with 40% milk.

## 2. Results and Discussion

### 2.1. Chemical Composition

The proximate chemical composition of beetroot plant parts is shown in (Table 1). There are no significant differences (*p* > 0.05) detected between peels and pomace regarding all the chemical parameters, this might be due to the difficulty of removing the thin peel from the pulp. Consequently, the juice also showed no significant differences (*p* > 0.05) with peels and pomace concerning total lipids, total sugars, and ash content. Interestingly, the leaves had the highest amount of protein (5.64%), which comprised a promising good source of protein. The obtained results were higher than that previously reported by Biondo et al. [8] where protein content was 3.81%. Furthermore, there was a discrepancy between the protein content of pomace (1.13%) in the present study and the other previous studies; It was much less compared to 45.53% by Shyamala and Jamuna [21], and slightly fewer than 1.6% as noted by Neha et al. [22]. These differences reasonably attributed to the different nitrogen content that resulted from the variations in nitrogen fertilization, the properties of the soil and other environmental conditions. It is also worth mentioning that leaves and stems exhibited the highest total lipids content (0.43 and 0.41%), respectively. These findings disagreed with Biondo et al. [8] who reported 0.78%, with a high concentration of the essential fatty acid (linolenic acid). Whereas, pomace contain fewer lipids portion (0.15%) which was in agreement with the USDA value (0.17%) described by Neha et al. [22], but it was lower than 0.31% as evaluated by Shyamala and Jamuna [21]. The sugar content in pomace and peel was 8.79% and 8.4%, respectively, which was slightly less than 9.56% in beetroot plant as reported by Neha et al. [22]. The sugar content in leaves was 0.44% which was nearly twenty times less than the sugar content in the pomace. These results were much less than 3.98% evaluated by Biondo et al. [8]. On the other hand, the juice sugar content was 4.8% which was higher than Kazimierczak et al. [23], who reported that the sugar content was 3.33% in the juice. The crude fiber was 2.6, 1.97, 2, and 2.15 in peels, pomaces, stems, and leaves, respectively. The value in pomace was less than the expected value (2.8%) as in USDA nutritional data reported by Neha et al. [22], and 35.53% as reported by Shyamala and Jamuna [21]. It might be the filtration of pomace during preparation decreased its fiber content. The obtained results revealed that the beetroots plant is a promising nutritional source for macronutrients which make it a good source for supporting several kinds of food products.

### 2.2. Minerals Content

The analysis of minerals content revealed that, juice contained the highest level of all detected minerals, except for calcium (412.52 mg/100 g) and magnesium (217.6 mg/100 g) (Table 1). At the same time, it is worth mentioning that all samples in the present study were high in potassium content ranged from 635 mg/100 g to 3053.7 mg/100 g; which is known to manage the blood pressure and cardiovascular system on the long-term usage [24,25]. In addition, the juice showed the highest value of iron (911.65 mg/100 g) which plays a role in anemia prevention [22]. On the other hand, Phosphorus in pomace was 41.02 mg/100 g which is in a variation range of (32.43–256 mg/100 g), and close to 40 mg as reported by Neha et al. [22], but less than 293.81 mg/100 g detected by Shyamala and Jamuna [21]. Results also revealed that potassium in leaves was 2196.1 mg/100 g which agreed with Biondo et al. [8] being 2078.4 mg/100 g. Potassium in pomace were 1971.6 mg/100 g which is six times higher than the USDA result reported by Neha et al. [22]. The highest amount of calcium found in leaves (1200 mg/100 g), which was significantly higher than that reported by Biondo et al. [8] being 186.46 mg/100 g. Although, pomaces had the lowest calcium content (154.92 mg/100 g), it was higher than that reported by Neha et al. [22] being 16 mg/100 g. Iron is considered one of the most crucial minerals, as it has a vital role in anemia treatment. The highest amount of Fe was in juice (911.65 mg/100 g), followed by peels (121.19 mg/100 g) and pomaces (99.19 mg/100 g). This value in pomaces was much higher than that reported by Neha et al. [22] being 0.8 mg, and Shyamala and Jamuna [21] who reported 11.61 mg/100 g of Fe. But the resulted value in leaves (13.71 mg/100 g) was less than that reported by Biondo et al. [8] being 25.63 mg/100 g. The obtained results in the current study emphasized the considerable content of crucial minerals that are necessary for human health and confirmed the importance of the beetroots as a good source of micro-and macro-elements.

### 2.3. Extraction Yield

**Figure 1:**
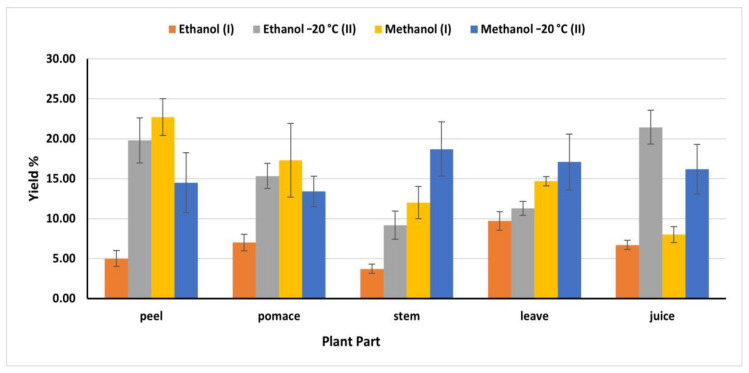
Yield of extraction of different solvents expressed in % of the dry matter; (I): IEM; (II): OEM at −20 °C.

Generally, phytochemicals extraction varied according to the solvent polarity and the nature of the extracted molecules, in addition to temperature [26]. Accordingly, using different solvents, temperatures, and extraction time resulted in various amounts of bioactive components (Figure 1). Ethanol (II) (OEM) exhibited the highest phenolic extraction capacity from juice (21.44%) compared to ethanol (I) (IEM) (6.70%). However, methanol (II) revealed the highest yield from stems and leaves, (18.70 and 17.10%) respectively, while methanol (I) extracted 12 and 14.70% from stems and leaves, respectively. On the other hand, methanol (I) extracted the highest bioactive compounds from peel and pomace (22.70 and 17.30%) respectively. The obtained yield from peel and pomace by methanol (I) was close to the amount obtained by ethanol (II) (19.80 and 15.34%) respectively (Figure 1). The extraction efficiency of phenolic compounds from different plant materials and different parts depends on the nature of the phenolic substances, polarity of the extraction solvents, time and temperature of the extraction.

### 2.4. Total Phenolic Content

**Figure 2:**
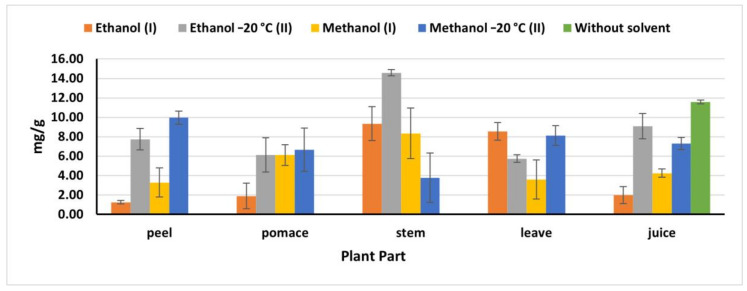
Total phenolics in beetroot extracts (mg gallic acid/g); (I): IEM; (II): OEM at −20 °C.

The natural juice had the highest phenolic content value (11.58 mg/g) compared to the phenolics obtained in the juice extracts (Figure 2), being nine times higher than the obtained amount by Kazimierczak et al. [23] (1.29 mg). It is also worth to be mentioning that (OEM) revealed a high phenolic extraction for stems and peels, while no significant differences were detected in the total phenolics obtained by IEM and OEM in pomace and leaves. As mentioned previously, OEM, particularly ethanol (II), exhibited high extraction efficiency with stems recording 14.58 mg/g, compared to 9.35 mg/g obtained by ethanol (I). Whereas in leaves, ethanol (I) extracted 8.54 mg/g, which is close amount as that extracted by methanol (II) (8.12 mg/g). The obtained results were less than the results reported by Biondo et al. [8]. Phenolic compounds extracted from pomace by methanol (II) were 6.66 mg/g which was significantly higher than that reported by Shyamala and Jamuna [21] (2.2 mg/g), while less than that obtained by Čanadanović-Brunet et al. [27] (376.4 mg/g). However, methanol II extracted 9.96 mg/g from peels, which was less than that value reported by Kujala et al. [28] (15.5 mg/g). This discrepancy in the extraction capacity of different methods in different parts and plant origins might have resulted to the environmental biotic and abiotic stresses, which influence the presence and distribution of the phenolic compounds in the plant [26] and also might be resulted to the essence and nature of the phenolics compounds and their concentration in the plant materials.

### 2.5. Total Flavonoids

**Figure 3:**
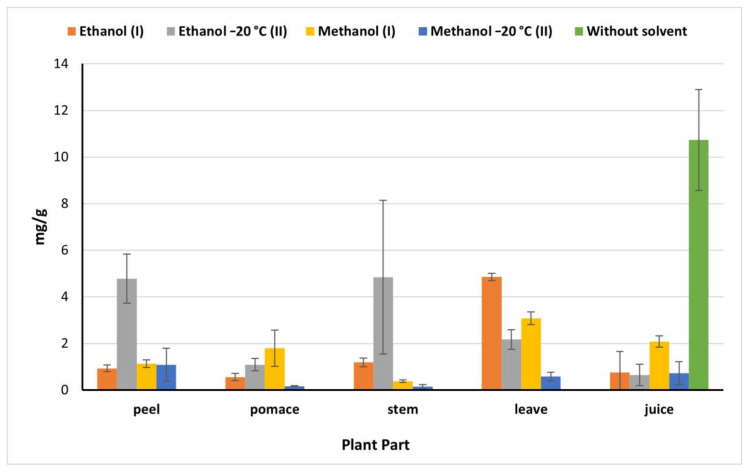
Total flavonoids of beetroot extract (mg catechin/g) based on the dry weight; (I): IEM; (II): OEM at −20 °C.

Juice showed a high flavonoids content (10.73 mg/g) compared to the flavonoids content determined in the other beet parts which declined significantly by 81% in the juice methanolic (I) (IEM) extract (Figure 3). However, this value was higher than the amount obtained by Kazimierczak et al. [23] (0.2 mg/g). While peels and stems ethanolic (II) (OEM) extracts exhibited the highest values of flavonoids being 4.78 and 4.84 mg/g, respectively, compared to 0.93 mg/g and 1.19 mg/g obtained by ethanol (I) peel and stems extract, respectively. Methanol (I) is more effective in the extraction of flavonoids in pomace (1.80 mg/g), which agreed with results of El-Beltagi et al. [29] being 1.54 mg/g, however, it was less than Čanadanović-Brunet et al. [27] being 253.5 mg/g. On the other hand, ethanol (I) extract exhibited the highest extraction ability of flavonoids from leaves (4.85 mg/g). Based on our findings, the beetroot is considered a good source of phenolic compounds and flavonoids which raises its nutritional value and benefits in food processing.

### 2.6. Betalain Content

**Figure 4:**
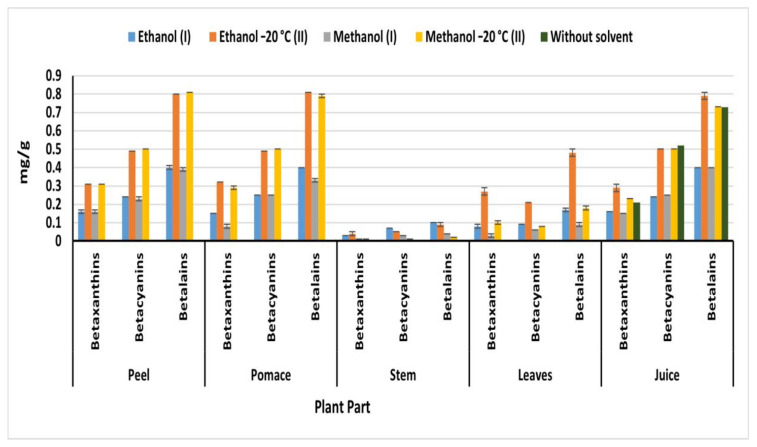
Betaxanthin, betacyanin, and betalain content of beetroot parts (mg/g); (I): IEM; (II): OEM at −20 °C.

Betalains are composed of red-violet betacyanins and yellow-orange betaxanthins [6]. The distribution of betalain pigment differs not only according to the beetroot parts, but also the extraction method (Figure 4). Generally, OEM extracted the highest betalain content compared to IEM in all parts. On the other hand, beetroot peels, pulps, and juice exhibited the highest betalain content compared to of leaves and stems, regardless of the used solvent. Methanol (II) OEM extracted the highest betalain content from peel (0.81 mg/g) compared to extracted by methanol (I) IEM (0.39 mg/g) (Figure 4). While ethanol (II) extracted the highest amount of betalain from pulp (0.81 mg/g), leaves (0.48 mg/g), and juice (0.79 mg/g) compared to 0.40, 0.18, and 0.40 mg/g obtained by ethanol (I) from pomace, leaves, and juice, respectively. The obtained betalain from beetroot peel, pulp, and juice was higher than 274.4 mg/kg detected in ethanolic extract (50%) of beetroot puree treated at 120 °C for 60 min [30]. Methanol (II) extracted 0.5 mg of betacyanin from peel and pulp, while 0.31 and 0.29 mg/g of betacyanin were detected in peel and pulp, respectively. The obtained result of betacyanin and betaxanthin was higher than 37.22 mg/100 g of betanin and 0.71 mg/100 g of vulgaxanthin I detected by Vulic et al. [20] in fresh beetroot pomace ethanolic extract. However, the obtained betalain from peel and pulp (0.81 mg/g) agreed with the betalain content of Redval and Forono varieties from Australia 853 and 826 mg/L, respectively [31]. On the other hand, Wruss et al. [31] detected another betalain concentrations ranged from 767 to 1309 mg/L in the different Austrian beetroot varieties. The findings in the current invistigation showed that the different parts of Egyptian beetroots are rich betalain sources particularly peels, pomace and juice. At the same time methanol is the best solvent for the extraction of betalain from different parts of beetroots.

### 2.7. HPLC Analysis for Total Phenolics

The HPLC chromatogram classified the phenolic acids and flavonoids in natural juice, and ethanolic (OEM) extracts of peels, leaves, and stems (Table 2). Chlorogenic (32.96 µg/g), gallic (25.19 µg/g), syringic (2.74 µg/g), and cinnamic acid (0.35 µg/g) were recognized in juice as phenolic acids in addition to catechin (93.56 µg/g). This result indicated not only the composition of juice but also the pomace. More phenolic acids and flavonoids were observed in peel extract, where catechin and gallic acid recorded the highest content than other parts being 184.50 and 137.23 µg/g, respectively. The obtained resulted compounds in peels were disagreed with the phenolics identified by Koubaier et al. [32] and El-Beltagi et al. [29]. The obtained results indicated that, the stems contained abundant phenolic compounds than the other parts, where rutin was the highest content followed by catechin being 241.58 and 149.9 µg/g, respectively. However, gallic and chlorogenic acids were not recognized in stem extract, which is disagreed with Koubaier et al. [32]. These differences in the level of phenolic compounds in different parts may refer to changes in growth conditions, especially availability which plays a crucial role in the accumulation level of phenolic compounds in different plant parts [33]. The higher of level of the phenolic compounds is responsible for the higher DPPH scavenging activity of all parts of the plant.

### 2.8. DPPH Scavenging Activity

**Figure 5:**
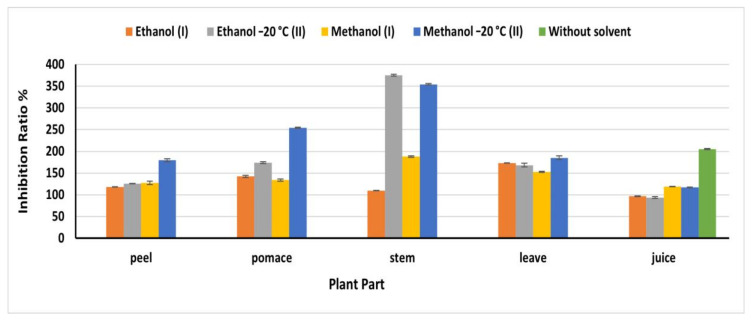
DPPH inhibition ratio% of beetroot extract based on the dry weight; (I): Instant Extraction method (IEM); (II): Overnight extraction method at −20 °C.

DPPH^•^ is a stable free radical that reduced in the presence of antioxidants, resulting in color changing from purple to yellow [34]. Methanol (OEM) extracts exploited high DPPH^•^ inhibition ratio compared to the other extracts, except for stem and juice (Figure 5). Where the natural juice revealed the highest antioxidant, activity compared to the extracts being 205.32%. Similarly, the ethanolic OEM extract of stems revealed the highest inhibition ratio (375.35%) at 91.8 mg/mL compared to 109.86% obtained from stem ethanol IEM extract, which agreed with the phenolic content detected in this extract. In leaves, methanol II extract revealed an antioxidant activity higher than that detected in the other extracts being 185.21% at 170 mg/mL. The inhibition ratios in peel and pomace methanolic OEM extracts were179.58 and 254.23%, respectively, which agreed with the phenolic content of this extract. The obtained results in the present investigation were higher than that reported by Vodnar et al. [19], who reported 45% in one gram of dried beetroot waste. In all cases, the beetroots extracts exhibited a high antioxidant power, which is greatly beneficial for human health and encourage us to fortify our food products with the beetroots extract.

### 2.9. Fermented Beet-Milk Beverage

#### 2.9.1. Total Viable Bacterial Count (TVBC) and Acidity

Probiotic products have proved to have at least 10^8^ colony forming unit (cfu) of prebiotic strains/mL juice [15]. Probiotic LA-5 and ABT-5 strains were used in this study to produce a probiotic beetroot- milk fermented beverage. The total viable cells of LA-5 and ABT-5 in addition to the beverage’s pH and acidity % during the fermentation were illustrated in Table 3. The milk was added to the beet juice to enhance the bacterial growth, as the LA-5 and ABT-5 numbers declined significantly after 6, 24, and 48 h of the fermentation of beet juice according to the preliminary studies. As noticed from the obtained results, pH value was reduced significantly at the end of the fermentation process from 6.6 in both LA-5 and ABT-5 fermented juices to 5.14 and 5.16, respectively (*p* < 0.05). The reduction of the pH was due to the production of lactic acid [35], which was confirmed by the % acidity of the juices. The acidity significantly increased from 0.41% of both samples at (zero time) to 1.84% and 1.94% at 6 h of fermentation for LA-5 and ABT-5 beverages, respectively (*p* < 0.05). *L. acidophilus* was increased significantly in LA-5 fermented beet-milk beverage after six hours of fermentation from 1.75 × 10^7^ to 1.25 × 10^10^ (*p* < 0.05). Similarly, the ABT-5 probiotic strain raised significantly from 2.37 × 10^7^ to 3.7 × 10^9^ after six hours of fermentation (*p* < 0.05). The obtained viable bacterial count in the present study was higher than 27.8 × 10^8^ resulted in beetroot juice after 72 h of fermentation with LA-5 strain as mentioned by Yoon et al. [35], and 7 × 10^8^ resulted from the fermentation of beet juice by LA-5 for 8 h [13]. Thus, adding 40% full-fat milk to the beetroot enhanced the bacterial growth and reduced the fermentation period.

#### 2.9.2. Chemical Analysis

Table 4 illustrates the chemical composition of control and fermented beet-milk beverages. Generally, no significant differences were detected in moisture, ash, lipids, and protein content of LA-5, ABT-5 fermented beverage, and the control sample (*p* > 0.05). Where the moisture content was ranged from 84.85 to 85.83 g/100 mL, total lipids were in a range of 3.03 to 3.06 g/100 mL which constituted about 21.5% of the beverage’ total dry matter. While the protein represented nearly 11.31% of the beverages dry matter, ranged from 1.48 to 1.68 g/100 mL. Ash content was about 1 g/100 mL. This result might be attributed to the short fermentation period, as the bacterial strains did not affect the nutrients in the beverage. On the other hand, carbohydrates constituted the highest part of all beverages, it was 8.61 g/100 mL for the control. However, it was reduced in the fermented beverages by nearly 30% as a result of the presence of the bacteria that converted the lactose into lactate to reach a value of 6.52 g/100 mL in LA-5 beverage, and 5.89 g/100 mL in ABT-5 beverage. As noticed in Table 4, no significant differences were detected between the fermented beet-milk beverages and the control beet-milk in magnesium, manganese, iron, copper, potassium, and sodium content (*p* > 0.05). Whereas, the calcium content was reduced after fermentation by 40% to reach 12.71 mg/100 mL in LA-5 fermented beverage, and 12.64 mg/100 mL in ABT-5 beverage compared to control (21.11 mg/100 mL). As noticed by Tang et al. [36], the calcium content was reduced after 12 h of fermentation of soymilk with *lactobacillus acidophilus* strains before being increased after 24 h of fermentation. Phosphorous content similarly was reduced after fermentation of the beverages by nearly 12%, being 53.71 mg/100 mL and 54.64 mg/100 mL in LA-5 and ABT-5 fermented beverages, respectively. The reduction of calcium and phosphorous could be due to the utilization of the probiotic culture of those elements in their growth [37]. On the other hand, fermentation recorded a slight increase in the zinc content of LA-5 and ABT-5 fermented beverages by 16% (0.27 mg/100 mL and 0.29 mg/100 g, respectively) compared to control, which might be resulted from releasing the metal from chelated complex compounds by bacterial activity and increasing its bioavailability [37].

#### 2.9.3. Phenolics Content and Antioxidant Activity

Table 5 shows total phenolics, flavonoids content, and % DPPH free radical scavenging activity of control and fermented beet-milk beverages. Fermentation of beet-milk beverage with LA-5 and ABT-5 probiotic strains raised the total phenolic content significantly compared to the control beverage. As total phenolics in LA-5 fermented beverage increased by 40% to reach a value of 17.51 mg/mL (Table 5). While a 31% increase in the total phenolics content was reported in ABT-5 fermented beverage to reach 16.38 mg/mL (*p* < 0.05). The increase in the total phenolics might be associated with the probiotic activity during the fermentation. The total phenolic content of fermented beverages could demonstrate the rise in the DPPH free radicals scavenging activity of LA-5 and ABT-5 beverages compared to the control, being 98.11, 97.89, 95.2%, respectively. On the other hand, no significant differences (*p* > 0.05) were determined concerning the flavonoid content of the LA-5 fermented beverages (9.91 mg/mL), ABT-5 fermented beverage (10.06 mg/mL), and the control sample (9.66 mg/mL).

#### 2.9.4. Sensory Evaluation

**Figure 6:**
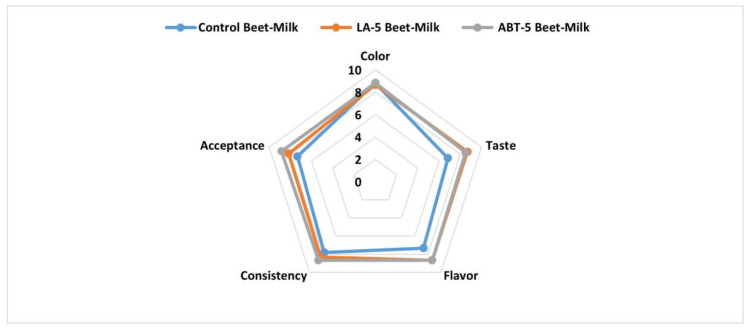
Sensory evaluation of control and fermented beet-milk beverages with LA-5 and ABT-5 probiotic cultures.

The data of the current investigation showed that, the fermentation process enhanced the taste and the flavor and consequently, the overall acceptance of LA-5 and ABT-5 fermented beverages compared to control beet-milk beverage. These results might be attributed to the formed lactic acid by probiotic strains [13]. On the other hand, no significant differences were detected in the acceptability of the fermented beverages and the control concerning color and consistency (Figure 6). So, the formulated fermented beverage-based-beetroots did not affect the sensory properties of the formulated functional beverage.

## 3. Materials and Methods

### 3.1. Materials and Reagents

Twenty-five kilograms of beetroot collected from Alexandria’s local market, Egypt. Fresh full cream milk was purchased from a local market in Alexandria (protein 2.6%, fat 3%, carbohydrates 4.6% calcium 97 mg/100 mL and phosphorus 79.61 mg/100 mL). Two freeze-dried lactic probiotic cultures; ABT-5 probiotic consists of (*Lactobacillus acidophilus LA*-*5*, *Bifidobacterium bifidum BB*-*12*, and *Streptococcus thermophilus*), and (*Lactobacillus acidophilus LA-5*) were obtained from Christian Hansen’s, Denmark. Absolute ethanol and methanol, chloroform, sodium hydroxide, sulphuric acid, boric acid, monopotassium phosphate, sodium carbonate, aluminium chloride and other commercial chemicals were supplied from Aljumhoria Company for chemicals, Alexandria, Egypt, Fine chemicals such as Folin-Ciocalteu, DPPH and phenolics standards purchased from Merk, Germany.

### 3.2. Preparation of the Beetroot Samples

Stems, and leaves were separated and washed, while the roots washed thoroughly to get rid of any soil residues. After peeling, the juice was extracted from the pulp. Peel, pomace, juice, leaves, and stems were used for further analysis. The plant parts were dried at 50 ± 2 °C for three days in (Wt-binder) drying oven; then each was milled until getting a fine powder by grinder (Kenwood FP691, UK). Afterward, the powder was packed into polyethylene bags as well as the extracted juice and stored at −20 ± 9 °C till used [21].

### 3.3. A Proximate Chemical Analysis

**Moisture content** was carried out using (Wt-binder) oven at 105 ± 2 °C for 24 h. till getting a constant weight of the samples. **The ash content** was determined by using a muffle furnace at 500 °C. **Crude fibers** was determined according to AOAC [38] by boiling the samples for 30 min with 1.25% of H_2_SO_4_, then NaOH after filtration and washing with hot water. The samples were dried in an oven, weighed (W1) and re-dried in a muffle till gray ash was formed and re-weighed (W2), then the crude fiber was calculated as g/100 g by the following Equation (1):

(1) $$\mathrm{Crude}\;\mathrm{fibers}=\frac{W1-W2}{\mathrm{Sample}\;\mathrm{weight}}\times 100$$

**Total fat** extracted with chloroform-methanol solvent according to Folch method [39]. **The protein content** was determined by the micro-Kjeldahl method according to Peach and Tracy [40]. **Total sugar** was calculated by difference according to the following Equation (2):

Total sugar content = 100 − (moisture + lipids + protein + fibers + ash)(2)

### 3.4. Minerals

Dried samples (1 g) of each wet-digested using conc. H_2_SO_4_-H_2_O_2_ mixture as described by Lowther [41]. Phosphorus content estimated by Vanadomolybdophosphoric yellow color method and the absorbance of the sample was measured at 405 nm. The concentration of phosphorus was determined by using monopotassium phosphate standard curve [42], Whereas the potassium content measured by the Backman flame photometer as described by Jackson [42]. Calcium, Magnesium, manganese, copper, iron, and zinc estimated by using the Inductively Coupled Argon Plasma (ICAP 6500 Duo, Thermo Scientific, Gloucester, UK), which standardized by 1000 mg/L multi-element certified standard solution, Merck, Germany.

### 3.5. Extraction Methods

The phytochemical compounds in the samples were extracted by the following two methods:

**Instant extraction method** (**IEM**): the extraction performed using ethanol (I) 70%, methanol (I) 80%, and water (I) as Vasconcellos et al. [43] with a slight modification, where each sample was vortexed with each solvent in a ratio of 1:10 (w/v) for 1 min, centrifuged for 10 min at 6000× *g*, and filtered. The pellet of each sample was re-extracted with the same solvent twice, and the filtrates combined before evaporating the solvent at 40 °C. The yield calculated as g/100 g, then the lyophilized samples were stored at −20 ± 9 °C till used.

**Overnight extraction method (at −20 °C) (OEM)**: Each sample was vortexed with methanol (II) 80%, and ethanol (II) 70% solvents in the same prior ratios and stored overnight at −20 ± 9 °C before extracting to increase the time of exposure of the plant part to the solvent for enhancing the extraction without being affected by the high temperature followed by the extraction of bioactive component as mentioned previously.

### 3.6. Total Phenolic Content (TP)

Total phenolic content was determined according to the Folin-Ciocalteu method as described by Vondar et al. and Raupp et al. [19,44]. 200 µL of the extract was mixed with 1 mL of 0.2N Folin-Ciocalteu reagent, and 800 µL of Na_2_CO_3_ (7.5%). The mixture incubated for 2 h in the dark at the room temperature, before reading the absorbance at 760 nm by using (Jenway 6405UV/VIS) spectrophotometer. The total phenolics expressed in mg/g as gallic acid equivalent based on the dry weight using a standard curve of gallic acid.

### 3.7. Total Flavonoids Content (TF)

Total flavonoids content was determined by aluminum chloride method as described by Čanadanović-Brunet et al. and Baba and Maik [27,45]. 1 mL of each extract was mixed with 4 mL dH_2_O and 0.3 mL 5% NaNO_2_ before incubating the mixture for 5 min. Afterward, 0.3 mL of 10% AlCl_3_ was added, and the samples were incubated for 6 min. Later, 2 mL of NaOH (1 mol/L) was added, and the volume completed with dH_2_O up to 10 mL. The mixture then incubated for 15 min before measuring the absorbance at 510 nm. The total flavonoid value expressed as mg/g of catechin on dry matter basis.

### 3.8. Betalain Content

Betalain content of beetroot parts extracts were estimated spectrophotometrically as described by Anand et al. and Castellanos-Santiago and Yahia [46,47]. Betacyanin and betaxanthin content were measured in the extracts at 535 and 483 nm, respectively by using (Jenway 6405UV/VIS) spectrophotometer. Betalain content was calculated as mg/g by the following Equations (3) and (4):

(3) $$\mathrm{Betacyanin}\;/\;\mathrm{Betaxanthin}\;(\mathrm{mg}/g)\;=\;\frac{A\times \mathrm{DF}\times \mathrm{MW}\times V}{\varepsilon \mathrm{LW}}$$

where: A is the maximum recorded absorption for betacyanins and betaxanthins, respectively, DF is the dilution factor, V is the extract volume (mL), W is the dried sample weight (g), and L is the path-length (1 cm) of the cuvette. The molecular weight and molar extinction coefficient (ε) of betacyanin are 550 g/mol, 60,000 L/(mol cm) in water, and of betaxanthin are 308 g/mol, 48,000 L/(mol cm) in water, respectively.

*Betalain content mg*/*g* = *betacyanin* (*mg*/*g*) + *betaxanthin* (*mg*/*g*) (4)

### 3.9. HPLC Profile of Phenolic Compounds

Phenolic compounds profile identified in natural juice, ethanolic (II) extract of peels, stem, and leaves by using an Agilent 1260 series HPLC with a multi-wavelength detector monitored at 280 nm. The injected 10 μL of each extract separated on a C18 column (4.6 mm × 250 mm i.d., 5 μm) at 35 °C, and a mobile phase consisting of water (A) and acetonitrile (B) at a flow rate 1 mL/min. Where the mobile phase was programmed consecutively in a linear gradient as follows: 0 min (80% A); 0–5 min (80% A); 5–8 min (40% A); 8–12 min (50% A); 12–14 min (80% A), and 14–16 min (80% A). The unknown compounds characterized by matching its retention time to the standard’s retention time.

### 3.10. DPPH Free Radical Scavenging Assay

DPPH assay was carried out according to Do et al. [34]. 0.5 mL of freshly prepared 0.3 mM methanolic DPPH mixed with 0.5 mL of the extract, then the mixture incubated at room temperature for 20 min. Afterward, the absorbance of the control (DPPH methanolic solution), and the samples measured at 517 nm. The inhibition ratio % was calculated by the following equation:

% Inhibition = (absorbance of control − absorbance of sample/absorbance of control) × 100

### 3.11. Preparation of the Fermented Juice

Beetroot juice was mixed with 40% full fat milk before being homogenized and heated at 80 °C for 20 min to mimic pasteurization step [16]. After heating, the mixture was cooled to 40 °C and aseptically inoculated with 0.2 gm/L of ABT-5 and 0.33 gm/L of LA-5 probiotic direct vat cultures for the preparation of ABT-5 juice and LA-5 juice, respectively. Afterwards, the inoculated juices were homogenized incubated at 37 °C for six h [48].

### 3.12. Total Viable Bacterial Count (TVBC)

The total viable bacterial count was determined in ABT-5 and LA-5 juices at the zero time, 3 and 6 h of fermentation. The viable cell count (CFU/mL) in the ABT-5 and LA-5 juice samples was counted in an MRS medium by using ten-fold serial dilution in peptone-water and a MRS standard plate count, the results were expressed as log of (cfu/mL juice) [35,48].

### 3.13. pH and Acidity Analysis

pH of the functional beverage samples was measured by a calibrated PH meter (Adwa, AD1030). The acidity was determined according to Kazimierczak et al. and Yoon et al. [23,35] by titrating the juices with 0.2N NaOH till the pH reached 8.2. The total acidity was expressed as % of lactic acid by using the following Equation (5):

% Acidity = (V × N × 0.09/W × 100)(5)

where: (V) is mL of NaOH, (N) normality of NaOH, and (W) weight of the sample.

### 3.14. A Proximate Chemical Analysis of Fermented Beverage

Moisture and ash content of LA-5 and ABT-5 fermented beet-milk beverages and beet-milk control were determined according to AOAC [38]. Total fat of beverages was estimated by Folch method [39], protein was determined by the micro-Kjeldahl method according to Peach and Tracy [40]. While the total carbohydrates were estimated by Phenol-Sulfuric acid method [49]. Mineral content was determined as described previously at **Section 3.4**.

### 3.15. Bioactive Components and Antioxidant Power of Fermented Beverage

Total phenolics, total flavonoids, and DPPH free radicals scavenging were determined as mentioned by Vodnar et al. [19], Baba and Malik [45], and Do et al. [34], respectively.

### 3.16. Sensory Evaluation

Taste, flavor, consistency, and the overall acceptance were estimated in the ABT-5 and LA-5 juice compared to the control juice by ten panelists (6 females and 4 males) in ages average between 25 to 60. The control juice was served to the panelists, followed by randomized, coded fermented beetroot-milk juices to assess the different sensory characteristics by recording a score of nine based on (1–9) Hedonic scale [50].

### 3.17. Statistical Analysis

All experiments were conducted in triplicates. SPSS program version 16 was used to determine the difference between means by one-way ANOVA test and univariate test for bioactive component yield, total phenolics, flavonoids, and DPPH assay. Means were compared by using Duncan’s test at (*p* < 0.05).

## 4. Conclusions

The current study was conducted to evaluate the nutritional quality of beetroots to ascertain its potential use for formulation of novel functional foods. Furthermore, using beetroot juice in the development of a probiotic beetroot beverage using LA-5 and ABT-5 probiotic strains and evaluate the effect of the fermentation on the nutritional value of the beverage. The discarded beetroot wastes are rich in phenolics and antioxidants rather than crude fibers and minerals. Where the leaves had significant amounts of fibers, and lipids. Additionally, consuming 100 g of dried leaves powder meets the daily calcium recommendations as described by FDA (1000 mg/day). Beet juice, peels, and pomace exceed the daily iron recommendations (18 mg/day), which is considered an excellent source for iron and function as anti-malnutrition and anemia. Stems shared high antioxidant activity due to the high presence of phenolic compounds. Adding 40% milk to beet juice enhanced the growth of the probiotic strains on fermented beet beverage. The fermentation process resulted in a high lactate formation, which in turns resulted in a better taste, flavor, and thickening the consistency. Moreover, the fermentation increased zinc content in addition to antioxidant capacity. Hence, the finding of the current study emphasized the health benefits of beetroot’ leaves and stems -which represent beetroots by-products-due to their high content of antioxidants and minerals. That is encourage utilizing beetroots by-products as a good source of novel food additives, food supplements and formulation of some novel functional foods.

## Figures and Tables

**Table 1:** Proximate chemical composition and minerals content of beetroot plant parts.

Sample Type	Peel	Pomace	Stems	Leaves	Juice
**Proximate analysis ^1^**					
**Moisture** %	86.3 ± 0.98 ^b^	86.8 ± 1.98 ^b^	91 ± 0.99 ^a^	90.7 ± 0.42 ^a^	92.9 ± 0.1 ^a^
**Total lipids** %	0.2 ± 0.04 ^b^	0.15 ± 0.04 ^b^	0.41 ± 0.02 ^a^	0.43 ±0.04 ^a^	0.16 ± 0.03 ^b^
**Protein** %	1.02 ± 0.1 ^c^	1.13 ± 0.18 ^c^	2.45 ± 0.2 ^b^	5.64 ± 0.28 ^a^	1.21 ± 0.14 ^c^
**Crude fibers** %	2.6 ± 0.12 ^a^	1.97 ± 0.23 ^a^	2 ± 1.41 ^a^	2.15 ± 0.42 ^a^	ND *
**Ash** %	1.48 ± 0.21 ^a^	1.16 ± 0.3 ^a,b^	1.27 ± 0.15 ^a^	0.64 ± 0.22 ^b^	0.93 ± 0.01 ^a,b^
**Total sugars** %**	8.4 ± 0.76 ^a^	8.79 ± 2.28 ^a^	2.87 ± 2.22 ^b^	0.44 ± 1.31 ^b^	4.8 ± 1.11 ^a,b^
**Minerals** (**mg**/**100 g**) **^2^**					
P	32.43 ± 0.01 ^b^	41.02 ± 0.74 ^b^	36.24 ± 7.42 ^b^	40.23 ± 0.83 ^b^	256 ± 12.01 ^a^
K	635 ± 134.6 ^c^	1971.6 ± 129.53 ^b^	2831.3 ± 242.26 ^a^	2196.1 ± 146.94 ^b^	3053.7 ± 75.97 ^a^
Ca	235.36± 89.24 ^c,d^	154.92 ± 20.46 ^d^	495.7 ± 71.74 ^b^	1200 ± 127.1 ^a^	412.52 ± 0.18 ^b,c^
Mg	311.44 ± 65.37 ^a^	116.4 ± 19.81 ^c^	48.22 ± 3.47 ^c^	58.02 ± 3.11 ^c^	217.6 ± 39.84 ^b^
Fe	121.19 ± 13.62 ^b^	99.19 ± 19.01 ^b^	1.29 ± 0.45 ^c^	13.71 ± 3.36 ^c^	911.65 ± 20.11 ^a^
Cu	1.95 ± 0.1 ^b^	1.32 ± 0.18 ^b,c^	1.65 ± 0.13 ^b^	0.17 ± 0.06 ^c^	6.32 ± 1.01 ^a^
Mn	5.19 ± 0.74 ^b^	4.73 ± 1.51 ^b^	0.10 ± 0.32 ^b^	0.89 ± 0.44 ^b^	27.30 ± 3.68 ^a^
Zn	3.81 ± 0.68 ^b^	1.77 ± 0.6 ^b^	2.03 ± 0.35 ^b^	1.98 ± 0.91 ^b^	319.03 ± 26.78 ^a^

Mean values in a raw having different superscript are significantly different at (*p* ≤ 0.05); ND * (Not detected); ** Total sugars calculated by difference; (^1^) proximate analysis parameters expressed in (g/100 g); (^2^) minerals content expressed in (mg/100 g).

**Table 2:** Phenolic acids and flavonoids identification of beetroot plant parts.

Sample/Compound	Juice	Peel	Leaves	Stem
**Phenolics** (µg/g)				
Gallic acid	25.19	137.23	5.54	ND *
Chlorogenic acid	32.96	7.52	7.27	ND *
Cinnamic acid	0.35	ND *	0.14	9.94
Ferulic acid	ND *	1.81	1.21	59.18
Caffeine	ND *	ND *	1.47	39.66
Coffeic acid	ND *	ND *	ND *	61.36
Syringic acid	2.74	20.73	ND *	50.85
Ellagic acid	ND *	ND *	ND *	87.56
Coumaric acid	ND *	4.11	28.97	59.90
Vanillin	ND *	ND *	ND *	70.44
**Flavonoids** (µg/g)				
Rutin	ND *	ND *	ND *	241.58
Naringenin	ND *	2.75	12.83	61.43
Propyl Gallate	ND *	ND *	0.67	18.18
4′,7-DihydroxyisoFlavone	ND *	ND *	0.79	20.33
Querectin	ND *	0.88	ND *	87.68
Catechin	93.56	184.50	22.31	149.91

ND * Not detected.

**Table 3:** PH, acidity, and viable cell count of LA-5 and ABT-5 beet-milk beverage.

Time (h)	PH	Acidity *	CFU/mL **
**LA**-**5 Beet**-**milk Fermented Juice**			
**0**	6.6 ± 0.01 ^a^	0.41 ± 0.01 ^c^	1.75 × 10^7^ ± 1.1 ^b^
**3**	6.17 ± 0.02 ^b^	0.59 ± 0.01 ^b^	5.45 × 10^7^ ± 0.28 ^b^
**6**	5.14 ± 0.03 ^c^	1.84 ± 0.05 ^a^	1.25 × 10^10^ ± 25.07 ^a^
**ABT**-**5 Beet**-**milk Fermented Juice**			
**0**	6.6 ± 0.01 ^a^	0.41 ± 0.01 ^c^	2.37 × 10^7^ ± 0.65 ^b^
**3**	6.16 ± 0.01 ^b^	0.61 ± 0.00 ^b^	6.5 × 10^7^ ± 0.28 ^b^
**6**	5.16 ± 0.05 ^c^	1.94 ± 0.06 ^a^	3.7 × 10^9^ ± 3.54 ^a^

Different superscripts are significantly different at (*p* ≤ 0.05); * Acidity expressed as % of lactic acid; ** CFU/mL: colony forming unit of probiotic culture/mL of beet-milk beverage.

**Table 4:** Proximate chemical composition of control beet-milk and fermented beet-milk.

Parameters	Beet-Milk (Control)	Beet-Milk (LAB-5)	Beet-Milk (ABT-5)
**Chemical Compositional Analysis** *			
**Moisture %**	85.83 ± 2.78 ^a^	84.85 ± 1.31 ^a^	85.67 ± 0.93 ^a^
**Total lipids %**	3.06 ± 0.06 ^a^	3.03 ± 0.04 ^a^	3.04 ± 0.08 ^a^
**Total protein %**	1.48 ± 0.11 ^a^	1.65 ± 0.03 ^a^	1.68 ± 0.04 ^a^
**Total carbohydrates %**	8.61 ± 2.9 ^a^	6.52 ± 1.43 ^b^	5.89 ± 0.67 ^b^
**Ash %**	1.00 ± 0.03 ^a^	0.98 ± 0.13 ^a^	1.1 ± 0.14 ^a^
**Minerals** (**mg**/**100 g**) **			
Ca	21.11 ± 1.29 ^a^	12.71 ± 1.99 ^b^	12.64 ± 3.31 ^b^
P	61.87 ± 1.23 ^a^	53.71 ± 0.7 ^b^	54.64 ± 1.58 ^b^
mg	14.6 ± 1.56 ^a^	14.02 ± 1.81 ^a^	13.32 ± 0.17 ^a^
K	121 ± 1.4 ^a^	120 ± 0.71 ^a^	119.3 ± 1.1 ^a^
Na	37.5 ± 5.79 ^a^	48.25 ± 4.17 ^a^	50.5 ± 0.7 ^a^
Fe	2.95 ± 0.31 ^a^	2.78 ± 0.47 ^a^	2.63 ± 0.49 ^a^
Mn	0.07 ± 0.03 ^a^	0.13 ± 0.01 ^a^	0.12 ± 0.02 ^a^
Cu	0.09 ± 0.1 ^a^	0.07 ± 0.02 ^a^	0.44 ± 0.55 ^a^
Zn	0.24 ± 0.31 ^b^	0.27 ± 0.02 ^a,b^	0.29 ± 0.0a ^a^

Different superscripts are significantly different at (*p* ≤ 0.05); * Calculated as g/100 gm wet matter; ** Expressed in mg/100 gm dry weight basis.

**Table 5:** Total phenolics, total flavonoids, and DPPH% free radicals scavenging activity of control and fermented beet-milk.

Beverage	TP *	TF **	DPPH % ***
Control Beet-Milk	12.48 ± 1.00 ^b^	9.66 ± 0.52 ^a^	95.2 ± 0.88 ^b^
LA-5 Beet-Milk	17.51 ± 0.16 ^a^	9.91 ± 0.37 ^a^	98.11 ± 0.93 ^a^
ABT-5 Beet-Milk	16.38 ± 1.17 ^a^	10.06 ± 1.00 ^a^	97.89 ± 1.00 ^a^

Different superscript is significantly different at (*p* ≤ 0.05); TP * (total phenolics), TF ** (total flavonoids) are expressed in mg/mL wet weight basis; DPPH % ***: % of free radical’s inhibition ratio.
